# Surgical Treatment in Ædema of Glottis

**Published:** 1847-08

**Authors:** 


*Surgical treatment in œdema of glottis.*—We quote the following from the proceedings of the N. Y. Medical and Surgical Society, as reported for the Annalist, July 15, 1837.— Ed. Buffalo Medical Journal.

Dr. Buck stated, that it had long ago occurred to him, that in cases of the œdema of the glottis, when suffocation seecemed imminent, that the patient might be relieved by incising the edges of the glottis and epiglottis. An opportunity having recently occurred, he had had recourse to the practice with decided benefit. The patient suffering under inflammation of the fauces, became affected with dyspnœa, attended with paroxysms of impending suffocation. On passing the finger into the fauces, the epiglottis could be felt enormously swollen and tense. The swelling of the glottis could likewise be distinguished. In the operation, a pair of curved, sharp pointed scissors was employed, and afterwards, a curved, sharp pointed bistoury, guarded to within a quarter of an inch of the point. The patient expectorated two or three spoonsful of blood, mixed with the secretions of the fauces, and expressed himself decidedly relieved. Besides the operation, the patient was bled, leeched, inhaled aqueous vapor, &c. He recovered.

Dr. Clark said, that he thought the suggestion of Dr. B., a highly important one; the œdematous swelling, in such cases being principally situated on the posterior surface of the epiglottis, and on the upper and posterior part of the glottis. In this last situation it had a valvular action, rising up during expiration, but closing, and thus impending inspiration. Indeed, the fact that the difficulty occurred only during inspiration, was characteristic of the disease Dr. C. thought the parts could be safely and easily reached, and that the operation afforded every promise of relief.

